# How Many Older Informal Caregivers Are There in Europe? Comparison of Estimates of Their Prevalence from Three European Surveys

**DOI:** 10.3390/ijerph17249531

**Published:** 2020-12-19

**Authors:** Aviad Tur-Sinai, Andrea Teti, Alexander Rommel, Valentina Hlebec, Giovanni Lamura

**Affiliations:** 1 Department of Health Systems Management, The Max Stern Yezreel Valley College, 1930600 Yezreel Valley, Israel; 2 School of Nursing, University of Rochester Medical Center, Rochester, NY 14642-8404, USA; 3 Institute for Gerontology, University of Vechta, D-49377 Vechta, Germany; andrea.teti@uni-vechta.de; 4 Epidemiology and Health Monitoring, Robert Koch-Institute, 10439 Berlin, Germany; RommelA@rki.de; 5 Faculty of Social Sciences, University of Ljubljana, 1000 Ljubljana, Slovenia; Valentina.Hlebec@fdv.uni-lj.si; 6 INRCA IRCCS—National Institute of Health and Science on Ageing, Centre for Socio-Economic Research on Ageing, 60124 Ancona, Italy; g.lamura@inrca.it

**Keywords:** informal caregiver, survey, harmonization, unpaid care

## Abstract

Informal caregivers are people providing some type of unpaid, ongoing assistance to a person with a chronic illness or disability. Long-term care measures and policies cannot take place without taking into account the quantitatively crucial role played by informal caregivers. We use the European Health Interview Survey (EHIS), the European Quality of Life Survey (EQLS), and the Study on Health and Ageing in Europe (SHARE) to measure the prevalence of informal caregivers in the European population, and analyze associated socio-demographic factors. This rate ranges between about 13 percent in Portugal and Spain, and more than 22 percent in Luxembourg, Belgium, and Denmark. It declines in older age groups and, on average, is lower in men than in women in all countries studied, and lower among the poorly educated compared to those with higher levels of education. However, large variance was observed in the average share of informal caregivers for most countries between the three surveys. Our findings, estimated through the three surveys, reveal common trends, but also a series of disparities. Additional research will be needed to enable policy makers to access a richer and more harmonized body of data, allowing them to adopt truly evidence-based and targeted policies and interventions in this field.

## 1. Introduction

### 1.1. Why Do We Need to Estimate the Number of Informal Caregivers in 2020?

Estimating the number of informal caregivers in ageing societies is an activity that is strategically important for a series of reasons. These include, in the first place, the consideration that any serious effort to plan and implement long-term care measures and policies—from cash-for-care schemes, to work-care reconciliation interventions, to the provision of in-kind services, just to mention a few—cannot take place without taking into account the quantitatively crucial role played in this sector by informal caregivers. This is due, on the one hand, to the very large number of people who take care, in unpaid form, of a person needing their support, a group whose size has been estimated to outnumber that of the formal caregivers operating as paid staff in the long-term care sectors [1,2].

On the other hand, there are also more “qualitative” aspects to be considered in this regard, resting on the cross-disciplinary evidence that the health-related and economic effectiveness of formal care interventions increases when a holistic approach is adopted, i.e., that formal and informal care are two indissolubly connected components to be delivered in an integrated manner [3,4,5].

While the above reasons refer more to macro-level considerations, there are further, micro-level grounds that justify an increased attention into quantifying, as precisely as possible, the number of informal caregivers in our societies. While informal care entails many positive aspects, in terms of cohesion and mutual respect between generations, it has as well been identified per se as a risk factor for health and wellbeing [6,7,8,9]. Therefore, the availability of reliable information about the prevalence of this phenomenon is key also in terms of public health targeting, especially with regard to specific sub-groups of caregivers, such as, for instance, married women providing intensive care to dependent spouses [6].

The recent COVID-19 pandemic has shown even more dramatically the essential contribution of informal care, highlighting its protective role against the risk of contagion [10]. This evidence, however, should not lead to back-to-the-family policies, but rather stimulate investments in supporting what some have rightly defined as the “invisible front-line care” [11]. A further remark justifying the urgent need for rigorous estimations concerning the availability of informal caregivers in our ageing societies refers to the reliability of such estimations. This is crucial to ensure that the observed trends are interpreted correctly when developing plans to reorganize the existing long-term care supply, including the recruitment, training, and retaining of formal care staff. In this regard, however, diverging findings from different studies [12,13,14] seem to suggest that a comparative approach may be useful. Using diverse sources as a basis to extract averages, or using similar indicators to approximate the number of informal caregivers, may indeed contribute to prevent biased results (partly depending, also, upon the definition or methodology used) that might jeopardize the correct planning or implementation of a long-term care provision.

In light of the above considerations, after the identification of what different institutions and stakeholders understand under the term “informal caregiver”, in the following we analyze the comparability of data concerning the prevalence of informal care, as provided by existing cross-national and international sources in health, social, and long-term care. Attention will be paid to examine which informal caregivers’ features are considered by these sources, moving from basic factors affecting the availability of informal care across countries such as gender, age, and education. The main research question underpinning this approach is the following: what is the prevalence of informal care in Europe, and which factors are associated with it? In the methodological section, details on the surveys considered for this study will be elucidated, highlighting the reasons that have led to their choice, and illustrating their core characteristics and the variables selected for our analysis. A description of the adopted analytical strategy, of the achieved findings, and a discussion of their implications complete this article.

### 1.2. Definition of Informal Caregiver

In the scientific and grey literature, there is no agreement on the definition of “informal caregiving”. To capture the core meaning of this concept, it might be therefore useful to briefly review both the official definitions adopted by relevant institutions and civil society stakeholders, and those more frequently reported by the scientific community.

As for the former group, it includes crucial stakeholders in the long-term care sector include the World Health Organization (WHO), the United Nations (UN), the Organization for Economic Cooperation and Development (OECD), and a few international caregiver organizations and federations. Each of them adopts a partly different definition of informal caregiving and informal caregiver, as illustrated in the following:

WHO: although this organization often distinguishes between formal and informal care in its publications, it does not provide its official definition of “informal caregiving” nor of what is meant by “informal caregiver.” To some extent, the WHO provides global estimates of the direct and indirect costs of informal care for individual diseases (e.g., dementia) [15]; however, the concept of informal caregiving underlying these estimates remains only generally defined as an “unpaid” task;
UN: the definition of the United Nations is somewhat more precise and comprehensive [1]. It points out the diversity existing across regions and legislations in describing and using this concept, and remarks its overlaps with the concept of “family caregiver”. For the UN, “informal caregiving” primarily stands for all non-professional care provided—by choice or by default—by family members (i.e., next of kin), friends, neighbors, or other persons caring for people with long-term care needs at all ages, usually in private households;
OECD: among its multi-faceted interests, this organization regularly deals with “informal caregivers” in its annual publication “Health at a Glance” [16], where they are defined as those who “provide care to friends or family members, or may do so as part of non-contractual voluntary work”. In some of its publications [17], however, the concept is also sometimes used to describe “undeclared or illegal caregivers who receive a salary or compensation from the care recipient, but do not have an official contract with them and are not registered with relevant social security offices”. This terminology tries to capture an increasing world-wide phenomenon of (primarily migrant) care workers hired by private households to support them in the daily care of dependent family members, but it risks creating confusion. Therefore, it is to be welcomed that, for reporting purposes, the OECD considers as “informal caregivers” only family members, friends, or uncompensated volunteers who provide support to care recipients on a regular basis, and who do not receive cash or other benefits beyond those intended to support caring activities;
IACO: the International Alliance of Carers Organizations is an umbrella organization that advocates for caregivers at an international level, and is based in Washington DC (USA). While it refrains from a uniform definition of informal caregivers in order to take into account cross-national differences, it uses this concept by referring typically to persons who provide a minimum of 24 h of unpaid care per week over an average period of five years [18];
EUROCARERS is the European Association of carers organizations (as well as research and development organizations) that aims to bring together all countries based in the European Union (EU) for a common goal: to promote an evidence-based advocacy for informal care in Europe. EUROCARERS defines an informal caregiver as “a person who provides—usually—unpaid care to someone with a chronic illness, disability or other long lasting health or care need, outside a professional or formal framework” [19].

Similarly, the scientific community is characterized by a variety of studies, differing from each other in defining who qualifies as a caregiver, as well as for the methods used to measure and confirm the types of care provided [20,21]. In some studies, caregivers are limited to the co-residing spouses of persons with dementia who report providing some informal care [22,23]. Other studies merely refer to caregivers as those who provide help to someone needing support with one or more activities of daily living (ADLs) or instrumental activities of daily living (IADLs) [24,25]. Other surveys have defined caregiving by asking specific questions about the provision of assistance to a family member with a chronic illness or disability [20]. Overall, it can be stated that the most frequently occurring defining characteristics of an informal caregiver typically refer to a person who provides some unpaid, ongoing assistance with ADLs or IADLs to a person with a chronic illness or disability [20,26].

In light of the definitions illustrated above, it can be concluded that, taking into account both a societal and a scientific perspective, the following essential characteristics can be used to define an informal caregiver, as someone who provides care (1) at least weekly (2) to someone with a chronic illness, disability, or other long-lasting health, social or long-term care needs, (3) as part of an unpaid non-contractual voluntary work outside a professional or formal framework.

### 1.3. Comparability of Cross-Nationals vs. International Sources

Estimating the prevalence of informal caregiving is not an easy task. Available data is often neither based on a uniform reference year nor common data sources. Single country estimates also differ depending upon the data source, the survey wave, or the definition of informal caregiving used. Limiting ourselves to the prevalence rates calculated for the European countries and the US, that are most frequently cited in the literature, and to the age group for which data are more easily available, Table 1 shows figures broken down for the main adult population and for those aged 50 years and older.

In summary, it can be inferred that, despite partly heterogeneous data, there seem to be some convergence for the US towards a prevalence rate of informal caregivers reaching approximately 17% of the entire adult population, which drops to 7% among those aged 50 years or older. In Europe, the picture seems to suggest an opposite scenario, as the proportion of informal caregivers increases with age. While the prevalence of informal caregiving within Europe’s adult population confirms the share of 17.0% already observed for the US, in the age group of the over-50 s the same rate ranges between 13.5 and 25.6%.

Concerning the three surveys analyzed in this paper (see next section for the criteria followed in selecting these surveys), the prevalence rates reported by the literature at European level vary from 17% in the adult population, as measured by the European Quality of Life Survey (EQLS) [2], up to 25% for informal caregivers aged 50 and over, as estimated by the Survey of Health, Ageing and Retirement in Europe (SHARE) [29]. To our knowledge, so far, no estimations have been published using the European Health Interview Survey (EHIS) in this regard—neither by Eurostat (which coordinates this study), nor by other scientific analyses based on this dataset—even though this indicator has been available already from the second wave of this survey [30,31].

In light of this, in the following, we will compare estimations of the prevalence rate of informal care in Europe—understood as an individual task, and not as part of an organizational effort, such as that provided within the activity of voluntary organizations—by using the data provided by the three mentioned data sources: EQLS, EHIS and SHARE. Our focus will be on the supply side, i.e., we will be using data reflecting the informal care provided to others in need, and not data capturing the perspective of care recipients (who receive informal care). Divergent results will then be discussed, and possible explanations for these differences will be illustrated, in order to formulate suggestions to possibly achieve more comparable or convergent findings across different surveys using similar definitions in the future.

## 2. Measures and Methods

### 2.1. Choice of Surveys

The study is based on a quantitative approach using three international surveys: the European Quality of Life Survey (EQLS), the European Health Interview Survey (EHIS), and the Survey of Health, Aging and Retirement in Europe (SHARE). They represent the only surveys delivering regular data on the investigated topic at a European level.

EQLS is a monitoring tool that covers all twenty-eight EU member states and sets out to capture quality of life in multiple dimensions. Initiated in 2003 and reiterated in 2007, 2011, and 2016, it documents European citizens’ living conditions and social situation. It includes subjective and objective measures: reported attitudes and preferences, as well as resources and experiences. It covers the population aged 18+.

EHIS is a general population survey that yields statistical information on health status, health determinants, and healthcare activities in the EU. It aims to provide harmonized and high-comparability statistical data across the EU member states, supporting the monitoring of health policies on social inclusion and protection, health inequalities, and healthy aging. The survey was initiated in 2006 and reiterated between 2013 and 2015 in the twenty-eight EU member states, as well as in Iceland. Its general coverage is the population aged 15 and over, living in private households.

SHARE seeks to better understand the dynamics of the growing population of persons aged 50+ and to provide a research infrastructure for public policymaking on behalf of the aging population [32,33]. The data collected in SHARE provide a unique instrument with which to compare the health, economic situation, and welfare of older people in different European countries over time [34,35]. The survey—a multidisciplinary, cross-national bank of micro data on health, psychological, and economic variables [36,37]—was initiated in 2004 and reiterated in 2006, 2008, 2011, 2013, 2015, and 2017.

### 2.2. Main Characteristics of the Three Surveys: Choice of Variables

By using these three international surveys, we are able to compare the 50+ population that each survey investigates. Among the countries that were examined in each of the surveys, fifteen countries that participated in all three surveys were tested: Denmark, Luxembourg, Greece, Austria, Croatia, Germany, Italy, Poland, Slovenia, Czech Republic, Estonia, Portugal, Spain, Belgium, and Sweden. As to the time point of data collection, to make the measurements conducted in the different surveys as comparable as possible, we chose the data gathered in EQLS in 2016, EHIS in 2014, and SHARE in 2015. Each of these surveys has a set of questions dealing with informal care provision. By means of these questions, the main outcome of this study—i.e., “providing informal care”—has been defined as follows.

The definition in EQLS is based on the following question: “In general, how often are you involved in any of the following activities outside of paid work?”. The possible answers are: (a). Caring for and/or educating your children; (b). Caring for and/or educating your grandchildren; (c). Cooking and/or housework; (d). Caring for disabled or infirm family members, neighbours or friends under 75 years old; (e). Caring for disabled or infirm family members, neighbours or friends aged 75 or over. The variable “providing informal assistance” was defined as a dichotomous variable that receives the value of 1 if the respondent states to be involved in “d” or “e”.

The definition in EHIS is based on the following question: “Do you provide care or assistance to one or more persons suffering from some age problem, chronic health condition, or infirmity at least once a week?” Respondents are explicitly instructed to exclude any care provided as part of his or her profession. The variable “provides informal assistance,” was defined as a dichotomous variable that receives the value of 1 if the respondent answered that they gave assistance.

The definition in SHARE is based on two questions: the first was: “In the last twelve months, have you provided any kind of assistance listed on this card to a family member from outside the household, a friend or a neighbour”?; the second was: “Let us now talk about help within your household. Is there someone living in this household whom you have helped regularly during the last twelve months with personal care, such as washing, getting out of bed, or dressing?”. The question does not include looking after one’s own grandchildren. The variable “provides informal assistance” was defined as a dichotomous variable that receives the value of 1 if the respondent answered that they gave assistance (within the household or elsewhere) and 0 if not.

In addition to information about “giving informal assistance,” in EQLS and EHIS information is available about how intensively the assistance is provided, expressed in hours per week. The intensity of informal assistance is out of the scope of this paper, but a follow-up analysis comparing more in-depth the two surveys, with regard to this specific topic, is planned in the future.

### 2.3. Analytical Strategy

The study analyses the frequency among people aged 50+ of providing informal assistance to adults in need of care, by comparing the range of European countries included in all three surveys. To this purpose, a preliminary step consisted in filtering only the proportion of respondents belonging to the age group of those over 50, as this age limit characterizes the sample of SHARE. We then compared the overall prevalence of informal caregivers across countries, and differentiated the findings according to gender, levels of education, and age groups, since these represent the only comparable variables available in all three surveys. This allowed us to examine trends and disparities as a function of some of the main socio-demographic determinants.

As for weighting, when analyzing EHIS a weighting factor was applied by taking into account unit’s probability of selection, non-response and over-sampling or under-sampling of certain population groups, and adjusting the sample to external information on sex, age, region, and education, relating to the distribution of persons in the target population [28]. The sampling design weights of the SHARE and EQLS are based on the basic principles of probability-sampling with maximal population coverage ([38,39], respectively).

## 3. Results

**Figure 1:**
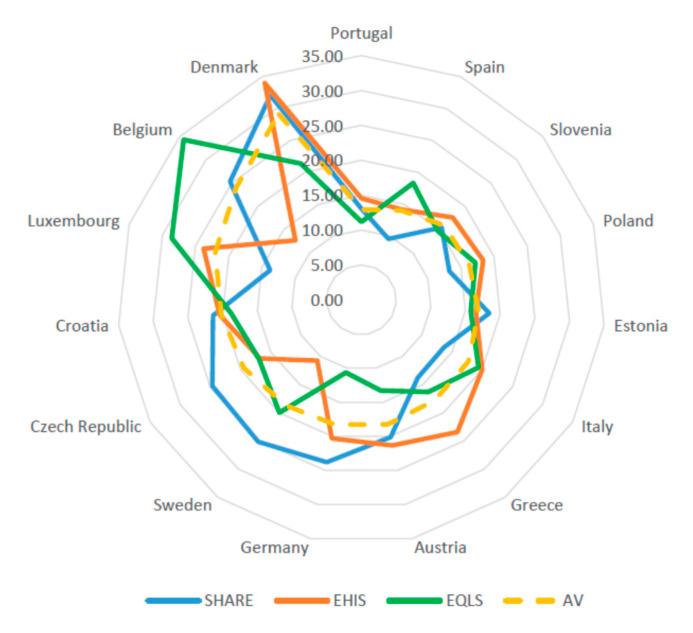
EHIS, SHARE and EQLS: Informal Caregivers over 50 (weighted), (percent). SHARE = Survey of Health, Ageing and Retirement in Europe; EHIS = European Health Interview Survey; EQLS = European Quality of Life Survey.

The proportion of informal caregivers in the population aged 50+ across the European countries covered by the three surveys EHIS, EQLS, and SHARE is shown in Figure 1. This share reaches nearly 13 percent in Portugal and Spain; is close to 18 percent in Central European countries such as Austria, Germany, and countries with Mediterranean welfare regimes such as Italy and Greece; and exceeds 22 percent in Luxembourg, Belgium, and Denmark. Beyond the differences across countries, some variance can be observed also in the rates emerging for most countries between the three surveys. Marginal differences among the surveys occur in Portugal, Slovenia, Estonia, and Croatia. For Spain, Luxembourg, and Belgium, in contrast, the share of informal caregivers estimated in EQLS is significantly higher compared to the other surveys, whereas for Austria, Germany, and Denmark the value measured by SHARE and EHIS is significantly higher than that estimated in EQLS. The rate of informal caregivers reported by SHARE for Italy and Greece is significantly lower than that measured for the same countries in EHIS and EQLS, whereas for Sweden and the Czech Republic this value is significantly higher in SHARE than in the other surveys (Figure 1, Table 2).

**Figure 2:**
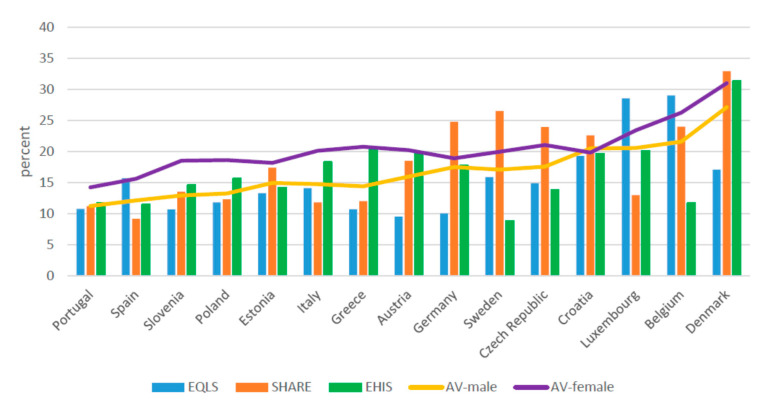
Informal Caregivers over 50 among males (weighted) (percent). EQLS = European Quality of Life Survey; SHARE = Survey of Health, Ageing and Retirement in Europe; EHIS = European Health Interview Survey; AV=Average.

**Figure 3:**
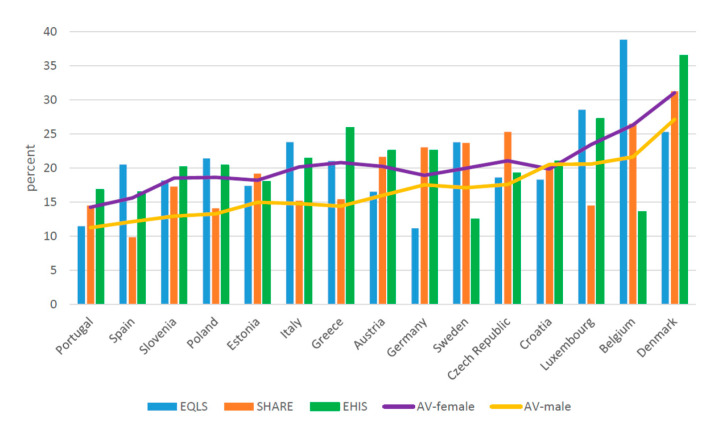
Informal Caregivers over 50 among females (weighted) (percent). EQLS = European Quality of Life Survey; SHARE = Survey of Health, Ageing and Retirement in Europe; EHIS = European Health Interview Survey; AV=Average.

**Figure 4:**
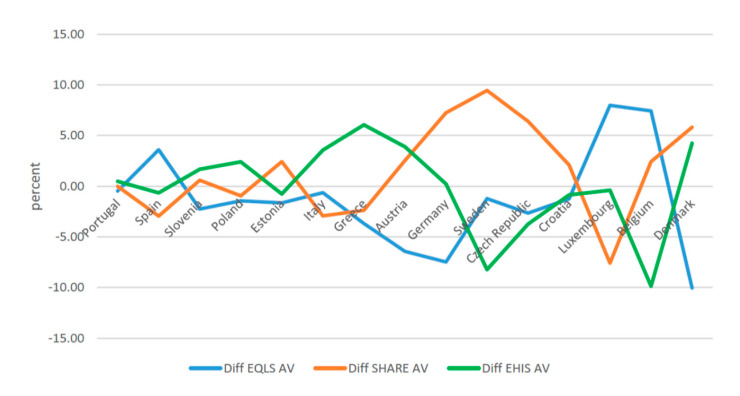
Difference between Informal Caregivers over 50 and average, males (weighted) (percent). EQLS = European Quality of Life Survey; SHARE = Survey of Health, Ageing and Retirement in Europe; EHIS = European Health Interview Survey.

**Figure 5:**
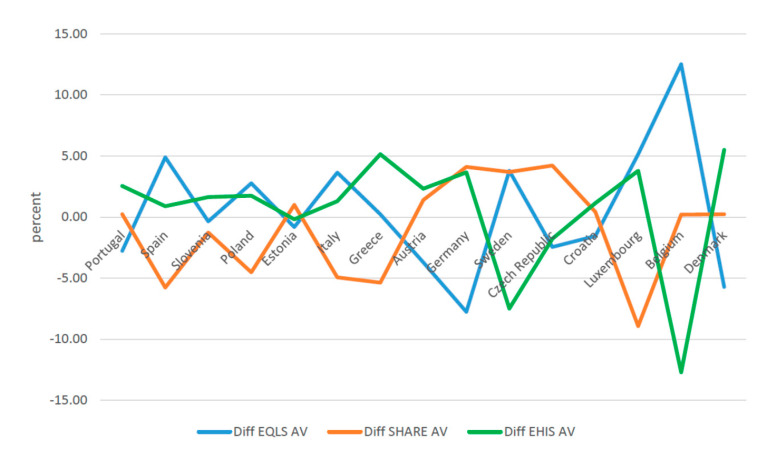
Difference between Informal Caregivers over 50 and average, females (weighted) (percent). EQLS = European Quality of Life Survey; SHARE = Survey of Health, Ageing and Retirement in Europe; EHIS = European Health Interview Survey; AV = Average.

When the estimated share of informal caregivers in the various surveys is distinguished by gender, again differences between the European countries are found (Figure 2 and Figure 3, Table 3). For both men and women, this rate is lowest in Spain and Portugal and highest in Luxembourg, Belgium, and Denmark. In all investigated countries, it is lower among men than for women. Among men, with the exception of Portugal, there are significant differences across the surveys. In Spain, Luxembourg, and Belgium, the rate reported by EQLS is significantly higher than that estimated in the other two surveys. In Poland, Italy, Greece, and Austria, the gap tilts in favor of EHIS, whereas in Estonia, Germany, Sweden, the Czech Republic, and Croatia, the highest rate is found for SHARE. Similar trends recurred for women in most countries. In EQLS, the share of informal caregivers was markedly higher for Spain, Italy, and Belgium compared to the other two surveys; in Portugal, Slovenia, Greece, and Denmark, the gap tilts in favor of EHIS, whereas in the Czech Republic it favors SHARE. Complementary material about differences in prevalence rates across countries on the basis of each survey, and the average rate calculated on the basis of the three surveys in each country, reveals considerable differences for Greece, Austria, Germany, Sweden, the Czech Republic, Luxembourg, and Denmark (Figure 4 and Figure 5).

**Figure 6:**
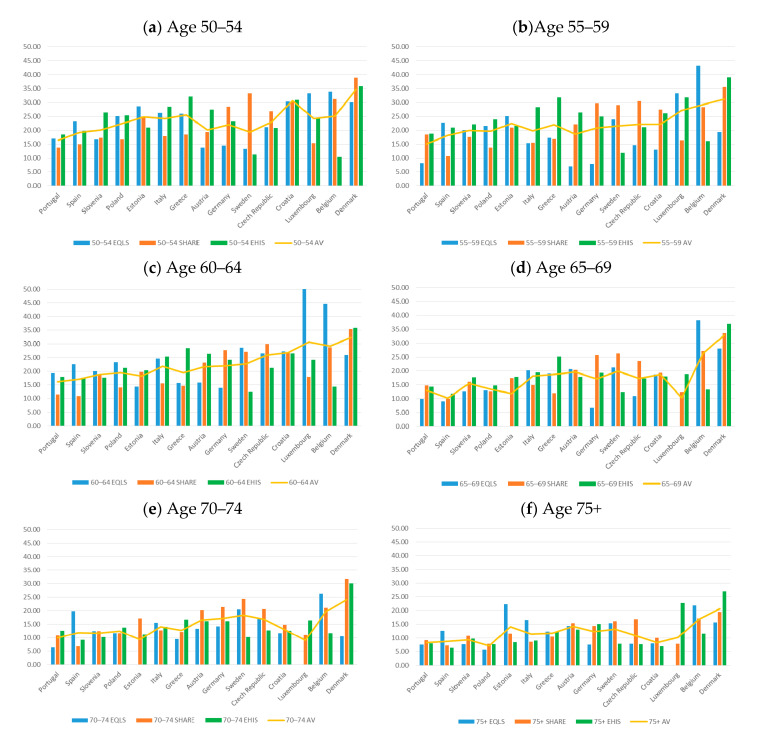
Informal Caregivers over 50, by age group (weighted) (percent) (**a**) Age 50–54 (**b**) Age 55–59 (**c**) Age 60–64 (**d**) Age 65–69 (**e**) Age 70–74 (**f**) Age 75+ (Small sample size regarding Slovenia, Estonia and Luxemburg; therefore results should be interpreted with caution). EQLS = European Quality of Life Survey; SHARE = Survey of Health, Ageing and Retirement in Europe; EHIS = European Health Interview Survey; AV = Average.

As for the role of age, it can be observed that the prevalence of informal caregivers declines in older age groups, particularly from the late fifties onward (Figure 6 and Table 4). Here, too, the share of informal caregivers varies between countries in every age group, as well as within each country, across the three surveys within the same age group. The proportion of informal caregivers is higher in EHIS than in the other two surveys in Austria, Denmark, Greece, and Luxembourg, for almost all age groups. Similarly, significant differences in favor of this survey were found among those aged 50–54 in Slovenia. The deviation is especially large between SHARE and the other surveys in the Czech Republic and Sweden, this being true for most age groups. A large difference in this direction was also found in the 70–74 year old cohort in Estonia and Croatia, and among those aged 75+ in Croatia. The rate of informal caregivers was found significantly higher in EQLS than in the other two surveys in Belgium, Estonia, and Spain, for most age groups. Furthermore, in the 60–64 cohort in Poland and Portugal and among those aged 75+ in Italy, this rate is higher in EQLS than in the two corresponding surveys.

**Figure 7:**
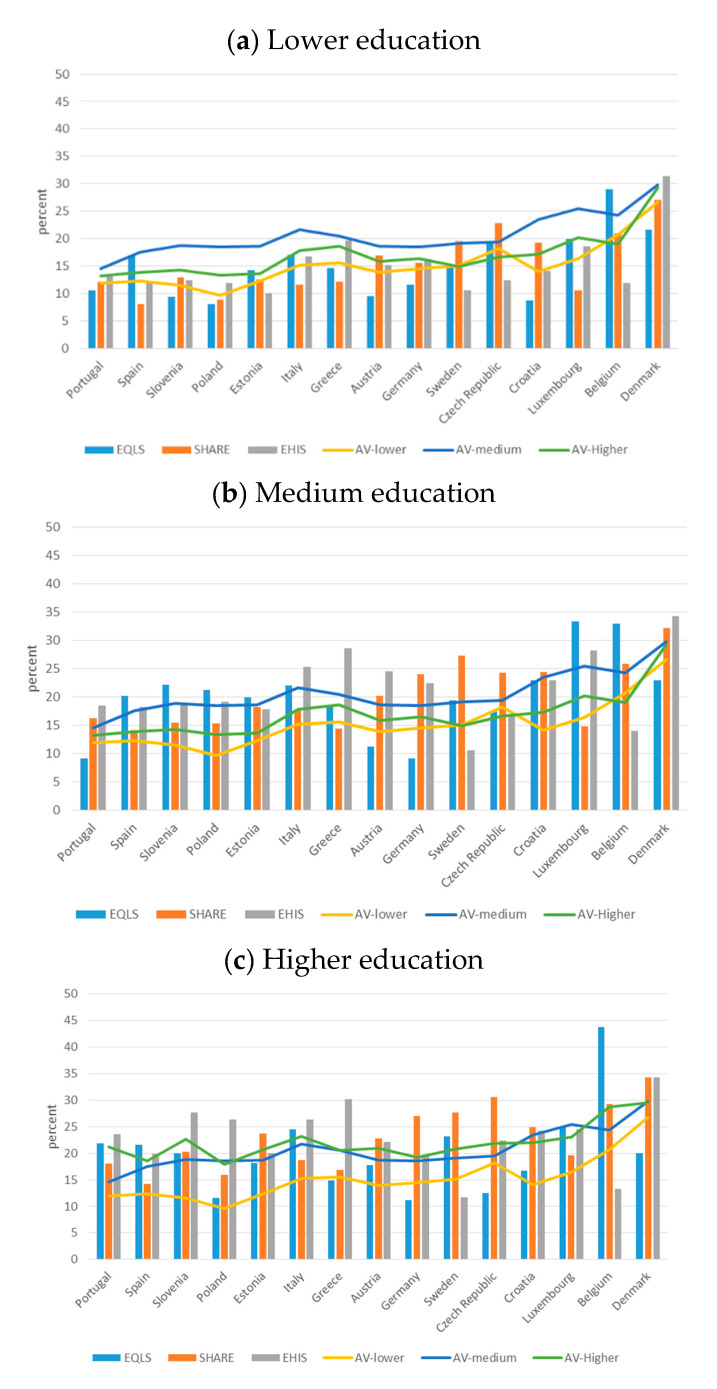
Informal Caregivers over 50, by education level (weighted) (percent) (**a**) lower education; (**b**)medium education; (**c**) higher education. EQLS = European Quality of Life Survey; SHARE = Survey of Health, Ageing and Retirement in Europe; EHIS = European Health Interview Survey; AV = Average

Finally, the prevalence of informal caregivers is on average lower among the poorly educated compared to those with higher levels of education (Figure 7, Table 5). The education categories are based on the International Standard Classification of Education (=ISCED)-2011 classification, where low level of education refers to ISCED-2011 0, 1 or 2; medium level of education refers to ISCED-2011 3 or 4; high level of education refers to ISCED-2011 5, 6, 7 or 8. For each educational level—low, medium, and high—variance for all countries was found. Furthermore, each country showed variance across the three surveys for the same educational levels. The rate of informal caregivers is markedly higher in EHIS than in the two other surveys for Poland, Italy, Greece, and Denmark, for most levels of education. This difference can be observed also for those with a medium-level education in Portugal and Austria, and for the well educated in Slovenia. The upward deviation from the other two surveys is especially large in SHARE for Sweden and the Czech Republic for most levels of education, as well as among the poorly educated in Croatia and the higher educated in Germany. The upward deviation from the other two surveys is particularly strong for EQLS in Spain, Estonia, and Belgian, for most education levels. This holds as well for respondents of medium education in Slovenia and Luxembourg.

## 4. Discussion

The main goal, underpinning the study presented here, was to understand whether the prevalence of informal caregivers aged 50+ in European countries can be determined unequivocally. To answer this question, we used the three main international surveys collecting data periodically on this topic in European countries: EHIS, EQLS, and SHARE. The increase in life expectancy [40,41] and the increase in the share of older people who age in place, remaining in the community even in very late life, justifies the importance of addressing this issue. Across countries, policy makers may find it helpful, if not even essential, to know how many informal caregivers live and operate in their countries, when they have to implement welfare and long-term care policies that recognise and build on the existing bulk of informal care delivered in their communities. Awareness about the size and role played by these key actors is crucial to design interventions in a way that enables them to be really helpful to those who, due to their functional hardships, need to count on a wide array of effective health, social, and long-term care services. Well-planned policies and measures can significantly influence family caregivers’ confidence in receiving the assistance and relief they need to address daily care needs. By doing so, they make the difference for the most vulnerable groups who are unable to secure such assistance, by establishing policies and measures that equip the care systems with more effective ways of providing the needed health and social services in a timely and qualitatively optimal way.

When we compared the three sources of information used for our study, we found that no single, converging truth exists. Our findings on the prevalence of informal caregivers, estimated through the three surveys, do reveal common trends, but also a series of disparities across the investigated countries. On the whole, differences among the surveys with regard to estimations concerning most Eastern European countries seem to be marginal. In the other countries, the differences among the surveys’ results do not systematically lean in favour of any particular survey. Supposing this divergence were related to the partially differing definitions used by each survey, we would have expected it to occur across all investigated countries, following a more or less similar pattern, but this has not been the case.

What is surprising—and this is probably one of the most interesting results emerging from the comparative exercise provided by this study—is that figures for some countries differ among the three surveys to a very large extent. This is true particularly for Belgium, Denmark, Germany, Greece, Luxembourg, and Sweden, all countries in which the difference between the lowest and highest figures suggested by the three surveys (Table 2) exceeds 10 percentage points (and even 20 points in the case of Belgium). Under such circumstances, evidence-based policy-making becomes rather difficult, given the high level of uncertainty connected with the choice of the one or the other figure. As this study could not go into an in-depth examination of the methodological aspects that might explain these discrepancies, this task will have to be investigated with by a future, more specific piece of research addressing this issue.

As to the other main results of our study, to some extent expectedly, the share of informal caregivers is in general higher among women than among men, whereas within each sex the different surveys report different prevalence rates. These fall in older age groups, particularly for individuals in their late fifties and older. Here again, as in the two cases described above, variance is found across countries with regard to each age group. Even when the three surveys are compared within the same age group, each country turns out to have a different prevalence of informal caregivers. In this regard, it should be acknowledged that we had to collapse all oldest age groups into a joint 75+ sub-group, thus partially losing information about oldest-old caregivers. Since in the coming years this is likely to become one of the most quickly growing segments of the caregiver population, we suggest that surveys like the ones analyzed in this study oversample older age groups, in order to have crucial information about this—for long-term care services very important—target group.

Education plays a crucial role, too. We found a lower prevalence among the poorly educated than among the middle and higher educated populations, with different countries reporting different rates of people serving as informal caregivers within each educational level, but also a variance with the same level of education in each country. These findings may be interpreted also in light of the well-known association between education and health [42], suggesting that the higher prevalence rates of caregiving found among the better educated population segments may be at least partly explained by their better health conditions. The current study, however, does not attempt to model population prevalence of informal caregiver in the different countries with regard to the socioeconomic characteristics, as this is beyond the aim of the current paper, whereas it is planned to deal with these aspects in future research efforts.

The current study has several limitations, which should be taken into account when interpreting the findings reported above. Foremost, even though the study focuses on persons aged 50+ who provide informal care, the questions formulated to capture this phenomenon are phrased differently in each of the three surveys in terms of the syntax used. It is not without consequences that each of the three surveys has its own specific goals: EHIS focuses primarily on health-related aspects; EQLS aims chiefly at understanding individuals’ quality of life; SHARE turns its main attention to assessing core aspects of older people’s health, economic status, and well-being. These partly divergent research goals are reflected by different sequences of questionnaire items in the three surveys. Moreover, it has been found in previous studies [43,44] that even when people are asked the same questions, their responses may be affected by questions previously posed to them on different topics. Consequently, even when the questions about aspects of informal caregiving are similar, the structure itself of the questionnaires may introduce some bias in individuals’ patterns of response.

Despite these limitations, the study presented here represents, to our knowledge, a major step forward in our evidence-based understanding of the role played by informal caregivers in our ageing societies. Recent years have seen major changes in the demographic structure, and a mix of different countries in terms of dependency and patterns of supply of and demand for health, social and long-term care services for the older population. These changes will be all the more salient in the years to come. Therefore, it will become increasingly crucial to understand and map, with the greatest possible accuracy, the size and main features of this population group, to better assess, design and implement the support services needed to facilitate their activity. The importance of this study traces to the very fact that it takes up for discussion the issue of the prevalence of informal caregivers. This has been undertaken with the hope that, by assessing the reliability and trustworthiness of existing statistics, suggestions could be formulated to improve them and that, by checking current indicators and metrics of informal caregiving and their impact in terms of empirical evidence across available sources, more carefulness may be invoked for welfare planning purposes.

In this regard, the main take-home message of this study can therefore be summarized as follows: given the large differences in the prevalence rates of informal caregiving reported for some countries by major studies in this field, data users should use current evidence deriving from these sources with caution. Data producers, on the other hand, should increase efforts to better understand why this divergence exists, and take action to possibly remove it, or more clearly explain the reasons behind such partly discording results. One contribution in this regard may come from adopting a more concise definition of informal caregiving in these surveys, so as to more precisely capture changes in these activities over time. Therefore, it can be concluded that additional research is needed to find solutions enabling policy makers across countries to count on a richer and more harmonized body of reliable data on such a fundamental component of our care systems, allowing them to adopt truly evidence-based and targeted policies and interventions in this field.

## Figures and Tables

**Table 1:** Mean prevalences of informal caregivers in international surveys.

Region	Prevalence		Source
	Caregivers 18+	Caregivers 50+	
US	16.8%		NAC/AARP Dataset, 2019 [27]
US		7.0%	HRS wave 13 (2015) [16]
Europe	17.0%		EQLS 2016 [2]
Europe		25.6%	SHARE/ELSA Meta-Analysis [28]
Europe		13.5%	SHARE 2017 [16]

NAC = National Alliance for Caregiving; AARP = American Association of Retired Persons; HRS = Health and Retirement Study; EQLS = European Quality of Life Survey; SHARE = Survey of Health, Ageing and Retirement in Europe; ELSA = English Longitudinal Study of Ageing.

**Table 2:** Informal Caregivers over 50, by survey (weighted) (percent).

	SHARE	EHIS	EQLS	Average
Portugal	13.05	14.56	11.18	12.93
Spain	9.56	14.19	18.31	14.02
Slovenia	15.49	17.62	14.75	15.96
Poland	13.28	18.37	17.20	16.28
Estonia	18.44	16.51	15.79	16.91
Italy	13.66	20.02	19.40	17.70
Greece	13.80	23.45	16.31	17.85
Austria	20.13	21.33	13.31	18.25
Germany	23.80	20.32	10.66	18.26
Sweden	25.14	10.76	20.00	18.63
Czech Republic	24.67	16.78	16.95	19.47
Croatia	21.38	20.42	18.75	20.18
Luxembourg	13.75	23.77	28.57	22.03
Belgium	25.31	12.75	34.28	24.11
Denmark	32.10	34.01	21.38	29.16

SHARE = Survey of Health, Ageing and Retirement in Europe; EHIS = European Health Interview Survey; EQLS = European Quality of Life Survey.

**Table 3:** Informal Caregivers over 50, by gender (weighted).

	Male			Female		
	EQLS	SHARE	EHIS	EQLS	SHARE	EHIS
Portugal	10.79	11.25	11.75	11.49	14.50	16.81
Spain	15.75	9.21	11.51	20.51	9.87	16.52
Slovenia	10.71	13.55	14.63	18.18	17.28	20.17
Poland	11.84	12.34	15.69	21.42	14.12	20.40
Estonia	13.33	17.40	14.22	17.39	19.20	18.03
Italy	14.15	11.87	18.35	23.81	15.22	21.46
Greece	10.74	12.03	20.47	21.02	15.44	25.93
Austria	9.57	18.53	19.90	16.54	21.64	22.57
Germany	10.06	24.79	17.77	11.18	23.04	22.60
Sweden	15.91	26.57	8.90	23.78	23.68	12.50
Czech Republic	14.93	23.99	13.85	18.63	25.29	19.30
Croatia	19.30	22.64	19.68	18.31	20.30	21.01
Luxembourg	28.57	13.00	20.20	28.57	14.51	27.22
Belgium	29.05	24.04	11.76	38.82	26.50	13.59
Denmark	17.11	32.95	31.38	25.30	31.27	36.52

EQLS = European Quality of Life Survey; SHARE = Survey of Health, Ageing and Retirement in Europe; EHIS = European Health Interview Survey; AV = Average.

**Table 4:** Informal Caregivers over 50, by age group (weighted) (Small sample size regarding Slovenia, Estonia and Luxemburg; therefore results should be interpreted with caution).

	50–54			55–59			60–64			65–69			70–74			75+		
	EQLS	SHARE	EHIS	EQLS	SHARE	EHIS	EQLS	SHARE	EHIS	EQLS	SHARE	EHIS	EQLS	SHARE	EHIS	EQLS	SHARE	EHIS
Austria	13.73	19.28	27.35	6.98	22.16	26.34	15.79	23.20	26.25	20.69	20.32	17.8	13.33	20.29	16.09	14.29	15.40	12.96
Belgium	33.85	31.20	10.36	43.14	28.22	15.96	44.68	28.79	14.24	38.30	27.13	13.21	26.32	21.07	11.59	21.74	17.13	11.53
Czech Republic	21.15	26.88	20.67	14.58	30.46	21.04	26.53	29.98	21.18	10.91	23.51	17.1	17.31	20.63	12.66	7.89	16.84	7.7
Germany	14.42	28.47	23.14	7.86	29.61	24.85	13.92	27.74	24.13	6.60	25.59	19.28	14.24	21.36	15.99	7.49	14.31	14.96
Denmark	30.00	38.84	35.69	19.23	35.54	38.86	25.93	35.43	35.68	28.00	33.57	36.87	10.53	31.71	30.09	15.63	19.40	26.91
Estonia	28.57	24.90	20.94	25.00	20.91	21.39	14.29	19.83	20.31	0.00	17.42	17.77	0.00	17.11	11.16	22.22	11.45	8.45
Greece	26.00	18.50	32.1	17.24	16.84	31.66	15.69	14.57	28.39	19.05	11.93	25.09	9.52	12.16	16.64	12.20	10.51	12.17
Spain	23.11	14.86	19.69	22.73	10.60	20.83	22.56	10.86	17.53	9.05	9.85	11.75	19.82	6.85	9.12	12.61	7.26	6.38
Croatia	30.43	30.39	30.93	13.04	27.42	26.02	27.27	26.93	26.42	18.75	19.29	17.9	11.76	14.86	12.58	8.00	10.10	6.88
Italy	26.22	17.89	28.45	15.36	15.51	28.18	24.62	15.51	25.34	20.15	14.90	19.44	15.58	12.77	14.07	16.46	8.55	9.03
Luxembourg	33.33	15.31	24.1	33.33	16.39	31.69	50.00	17.85	24.1	0.00	12.39	18.72	0.00	11.06	16.4	0.00	7.90	22.66
Poland	25.13	16.74	25.27	21.46	13.67	23.78	23.24	14.04	21.16	13.04	12.56	14.7	11.70	11.79	13.75	5.61	7.90	7.63
Portugal	16.95	13.74	18.44	8.16	18.46	18.64	19.35	11.38	17.75	9.84	14.75	14.29	6.38	10.78	12.6	7.58	9.15	8.04
Sweden	13.33	33.30	11.17	23.91	29.02	11.68	28.57	27.13	12.37	21.15	26.22	12.29	20.51	24.42	10.15	15.25	16.10	7.9
Slovenia	16.67	17.21	26.32	20.00	17.55	22.02	20.00	18.63	17.5	12.50	16.14	17.67	12.50	12.48	10.13	7.69	10.72	9.67

EQLS = European Quality of Life Survey; SHARE = Survey of Health, Ageing and Retirement in Europe; EHIS = European Health Interview Survey; AV = Average

**Table 5:** Informal Caregivers over 50, by education level (weighted).

	Lower			Medium			Higher		
	EQLS	SHARE	EHIS	EQLS	SHARE	EHIS	EQLS	SHARE	EHIS
Portugal	10.59	12.18	13.06	9.09	16.24	18.45	21.88	18.03	23.56
Spain	16.87	8.13	11.99	20.24	14.19	18.22	21.60	14.17	19.83
Slovenia	9.38	12.95	12.41	22.22	15.50	18.79	20.00	20.33	27.69
Poland	8.07	8.96	11.94	21.24	15.31	19.11	11.61	15.95	26.27
Estonia	14.29	12.66	10.05	20.00	18.19	17.91	18.18	23.71	19.99
Italy	17.10	11.72	16.84	22.08	17.56	25.41	24.47	18.63	26.36
Greece	14.71	12.26	19.74	18.37	14.47	28.65	14.81	16.78	30.12
Austria	9.52	16.97	15.18	11.21	20.18	24.54	17.78	22.76	22.14
Germany	11.68	15.57	16.34	9.13	24.10	22.41	11.13	26.94	19.76
Sweden	15.00	19.62	10.66	19.42	27.27	10.67	23.15	27.69	11.68
Czech Republic	19.35	22.86	12.46	17.41	24.32	16.66	12.50	30.59	22.40
Croatia	8.82	19.29	14.19	22.97	24.43	23.05	16.67	24.91	24.21
Luxembourg	20.00	10.64	18.71	33.33	14.85	28.22	25.00	19.59	24.48
Belgium	29.06	21.06	12.00	33.04	25.91	14.07	43.68	29.25	13.30
Denmark	21.62	27.11	31.44	22.95	32.18	34.33	20.00	34.21	34.22

EQLS = European Quality of Life Survey; SHARE = Survey of Health, Ageing and Retirement in Europe; EHIS = European Health Interview Survey; AV = Average.

## References

[1] United Nations (2017). Expert Group Meeting on Care and Older Persons: Links to Decent Work, Migration and Gender.

[2] EC (2018). Informal Care in Europe. Exploring Formalisation, Availability and Quality.

[3] Bryant J., Mansfield E., Boyes A.W., Waller A., Sanson-Fisher R., Regan T. (2016). Involvement of informal caregivers in supporting patients with COPD: A review of intervention studies. Int. J. Chronic Obstr. Pulm. Dis..

[4] Fiest K.M., McIntosh C.J., Demiantschuk D., Leigh J.P., Stelfox H.T. (2018). Translating evidence to patient care through caregivers: A systematic review of caregiver-mediated interventions. BMC Med..

[5] Heslin M., Forster A., Healey A., Patel A. (2016). A systematic review of the economic evidence for interventions for family carers of stroke patients. Clin. Rehabil..

[6] Bom J., Bakx P., Schut F., van Doorslaer E. (2019). The impact of informal caregiving for older adults on the health of various types of caregivers: A systematic Review. Gerontologist.

[7] Williams F., Moghaddam N., Ramsden S., De Boos D. (2019). Interventions for reducing levels of burden amongst informal carers of persons with dementia in the community. A systematic review and meta-analysis of randomised controlled trials. Aging Ment. Health.

[8] Wetzstein M., Rommel A., Lange C. (2015). Informal Caregivers-Germany’s Largest Nursing Service.

[9] Tur-Sinai A., Casanova G., Lamura G. (2019). Changes in the provision of family care to frail older people in familistic welfare states: Lessons from Israel and Italy. J. Aging Health.

[10] Chan E.Y.Y., Gobat N., Kim J.H., Newnham E.A., Huang Z., Hung H., Dubois C., Kei Ching Hung K., Lai Yi Wong E., Yeung Shan Wong S. (2020). Informal home care providers: The forgotten health-care workers during the COVID-19 pandemic. Lancet.

[11] Phillips D., Paul G., Fahy M., Dowling-Hetherington L., Kroll T., Moloney B., Duffy C., Fealy G., Lafferty A. (2020). The invisible workforce during the COVID-19 pandemic: Family carers at the frontline. Hrb. Open Res..

[12] Ciccarelli N., Van Soest A. (2018). Informal Caregiving, Employment Status and Work Hours of the 50+ Population in Europe. De Econ..

[13] Eurofound (2017). European Quality of Life Survey 2016: Quality of Life, Quality of Public Services, and Quality of Society.

[14] Verbakel E., Tamlagsrønning S., Winstone L., Fjær E.L., Eikemo T.A. (2017). Informal care in Europe: Findings from the European Social Survey (2014) special module on the social determinants of health. Eur. J. Public Health.

[15] WHO (2017). Global Action Plan on the Public Health Response to Dementia 2017–2025.

[16] OECD (2019). Health at a Glance 2019: OECD Indicators.

[17] OECD (2018). Care Needed: Improving the Lives of People with Dementia, OECD Health Policy Studies.

[18] IACO (2015). Global Organisations Working to Advance Carers Issues.

[19] EUROCARERS (2020). Definition of Informal Caregiving.

[20] Roth D.L., Fredman L., Haley W.E. (2015). Informal Caregiving and Its Impact on Health: A Reappraisal from Population-Based Studies. Gerontologist.

[21] Guida E., Barello S., Corsaro A., Galizi M.C., Giuffrida F., Graffigna G., Damiani G. (2019). An Italian pilot study of a psycho-social intervention to support family caregivers’ engagement in taking care of patients with complex care needs: The engage-in-caring project. BMC Health Serv. Res..

[22] Kiecolt-Glaser J.K., Preacher K.J., MacCallum R.C., Atkinson C., Malarkey W.B., Glaser R. (2003). Chronic stress and age-related increases in the proinflammatory cytokine IL-6. Proc. Natl. Acad. Sci. USA.

[23] Von Känel R., Dimsdale J.E., Mills P.J., Ancoli-Israel S., Patterson T.L., Mausbach B.T., Grant I. (2006). Effect of Alzheimer caregiving stress and age on frailty markers Interleukin-6, C-Reactive protein, and D-Dimer. J. Gerontol. Ser. A.

[24] Brown S.L., Smith D.M., Schulz R., Kabeto M.U., Ubel P.A., Poulin M., Yi J., Kim C., Langa K.M. (2009). Caregiving behavior is associated with decreased mortality risk. Psychol. Sci..

[25] Fredman L., Cauley J.A., Hochberg M., Ensrud K.E., Doros G. (2010). Mortality associated with caregiving, general stress, and caregiving-related stress in elderly women: Results of caregiver-study of osteoporotic fractures. J. Am. Geriatr. Soc..

[26] Schulz R., Tompkins C.A., National Research Council (US) Committee (2010). Informal caregivers in the united States: Prevalence, caregiver characteristics, and ability to provide care. The Role of Human Factors in Home Health Care.

[27] AARP (2020). Caregiving in the United States 2020 AARP.

[28] Kaschowitz J., Brandt M. (2017). Health effects of informal caregiving across Europe: A longitudinal approach. Soc. Sci. Med..

[29] Roll A., Litwin H. (2013). The exchange of support and financial assistance: Differences in exchange patterns and their implications for ageing well. Active Ageing and Solidarity between Generations in Europe. First Results from SHARE after the Economic Crisis.

[30] Eurostat (2013). European Health Interview Survey (EHIS Wave 2). Methodological Manual. Methodologies & Working Papers.

[31] Eurostat (2018). European Health Interview Survey (EHIS Wave 3). Methodological Manual. Methodologies & Working Papers.

[32] Tur-Sinai A., Shuldiner J., Bentur N. (2019). Sociodemographic inequality in joint-pain medication use among community-dwelling older adults in Israel. Health Soc. Care Community.

[33] Shuldiner J., Tur-Sinai A., Bentur N. (2020). Musculoskeletal pain medication use in middle age and older adults in 15 European countries and Israel. Pain Manag. Nurs..

[34] Ben-David N., Halperin D., Katz R., Lowenstein A., Tur-Sinai A. (2016). A Method for Estimating the Participation Rate of Elder Care. Theor. Econ. Lett..

[35] Lowenstein A., Katz R., Tur-Sinai A., Fernandez-Ballesteros R., Robine J.M., Benetos A. (2019). Intergenerational family relationships and successful aging. The Cambridge Handbook of Successful Aging.

[36] Katz R., Lowenstein A., Halperin D., Tur-Sinai A. (2015). Generational solidarity in Europe and Israel. Can. J. Aging La Rev. Can. Du Vieil..

[37] Tur-Sinai A., Lewin-Epstein N. (2020). Transitions in giving and receiving intergenerational financial support in middle and old age. Soc. Indic. Res..

[38] De Luca G., Rossetti C., Malter F., Malter F., Börsch-Supan A. (2015). Sample design and weighting strategies in SHARE Wave 5, In SHARE Wave 5: Innovations & Metodology.

[39] Vila J., Cervera J.L. (2014). Revision of the Weighting Strategy in the European Quality of Life Survey (EQLS).

[40] Litwin H., Tur-Sinai A. (2015). The role of the social network in early retirement among older Europeans. Work Aging Retire..

[41] Silverstein M., Tur-Sinai A., Lewin-Epstein N. (2020). Intergenerational support of older adults by the ‘Mature’ sandwich generation: The relevance of national policy regimes. Theor. Inq. Law.

[42] Zimmerman E., Woolf S.H. (2014). Understanding the Relationship between Education and Health. Discussion Paper.

[43] Story D.A., Tait A.R. (2019). Survey research. Anesthesiol. J. Am. Soc. Anesthesiol..

[44] Schwarz H., Revilla M., Weber W. (2020). Memory effects in repeated survey questions: Reviving the empirical investigation of the independent measurements assumption. Surv. Res. Methods.

